# Fast read alignment with incorporation of known genomic variants

**DOI:** 10.1186/s12911-019-0960-3

**Published:** 2019-12-19

**Authors:** Hongzhe Guo, Bo Liu, Dengfeng Guan, Yilei Fu, Yadong Wang

**Affiliations:** Center for Bioinformatics, Harbin Institute of Technology, 92 West Dazhi Street, Harbin, 150001 China

**Keywords:** Seed-and-extension alignment, Landau-Vishkin algorithm, Variation-aware read alignment

## Abstract

**Background:**

Many genetic variants have been reported from sequencing projects due to decreasing experimental costs. Compared to the current typical paradigm, read mapping incorporating existing variants can improve the performance of subsequent analysis. This method is supposed to map sequencing reads efficiently to a graphical index with a reference genome and known variation to increase alignment quality and variant calling accuracy. However, storing and indexing various types of variation require costly RAM space.

**Methods:**

Aligning reads to a graph model-based index including the whole set of variants is ultimately an NP-hard problem in theory. Here, we propose a variation-aware read alignment algorithm (VARA), which generates the alignment between read and multiple genomic sequences simultaneously utilizing the schema of the Landau-Vishkin algorithm. VARA dynamically extracts regional variants to construct a pseudo tree-based structure on-the-fly for seed extension without loading the whole genome variation into memory space.

**Results:**

We developed the novel high-throughput sequencing read aligner deBGA-VARA by integrating VARA into deBGA. The deBGA-VARA is benchmarked both on simulated reads and the NA12878 sequencing dataset. The experimental results demonstrate that read alignment incorporating genetic variation knowledge can achieve high sensitivity and accuracy.

**Conclusions:**

Due to its efficiency, VARA provides a promising solution for further improvement of variant calling while maintaining small memory footprints. The deBGA-VARA is available at: https://github.com/hitbc/deBGA-VARA.

## Introduction

An accurate and complete understanding of genetic variation is important in research on human disease [1–3]. A fundamental challenge of high-throughput sequencing (HTS) data analysis is accurate read alignment to one or multiple reference genomes. The mostly used procedure of HTS read alignment is to follow one haplotype at each reference site to map the reads. It is able to cause inherent mapping biases toward the standard reference, [4, 5], with great effect to subsequent analyses, such as variant calling [6], genotyping [7] and haplotype phasing [8, 9]. It is proven that with no existing variants, mapping reads directly to a reference genome can have a relatively high quality outcome in regions with low divergence [10]. However, complex regions consists a lot of biologically valuable single mutations and structural variants, e.g., the major histocompatibility complex (MHC) region that occurs on human chromosome 6, which includes the human leukocyte antigen (HLA) gene families. Analogous to the complex regions, other locations of high diversity also have strongly effect gene expression [11] and phenotypes [12], such as CpG islands [13], microsatellites [14], the HBB complex [15] and regions of genomic rearrangements [16].

These large genetic variations are likely to lead to lots of unmapped reads or low-quality mapped reads, resulting in poor quality on characterization of individual genomes and coverage fluctuations [17]. Moreover, it is still of great significance to characterize the variants that reside in novel sequences absent from reference genomes, for instance, Li et al.[18] revealed almost 5Mb novel sequences absent from reference genome by de novo assembly of individual genome. These data can be improved by the discovery of new variants in regions of segmental duplications and low complexity; A new set of variants created and a new DISCOVAR method was developed on this set to prove that 10% of the challenging genome harbors 30% of the variants, which was done by Weisenfeld et al. [10]. Also, Dilthey et al. [17] constructed a population reference graph (PRG) model which combined of 8 local assembled haplotypes in GRCh37 and other HLA alleles, in order to promote the performance of individual genome inference in the MHC region. Nam S Vo and Vinhthuy Phan [4, 19] experimentally demonstrated that incorporating given variants into read mapping can significantly made variant calling accuracy better with low-coverage data. To be more detailed, a 2-19% higher recall rate and a 9-34% higher precision rate of INDEL identification in that study, than that of GATK [20]. Beside of its better performance, the strategy is also be able to raduce the experimental cost.

Defining new data structures that can repersent different kinds of genome variants can be very challenging. Reads mapping to de Bruijn graphs, according to Limasset et al. [21], was suggested to be an NP-complete problem. In the meantime, a heuristic algorithm BGREAT was provided as a practical solution to improve the mapping capability compared to that of assembly-based strategies. It is non-trivial to identify optimal mapping candidates because of the explosively growing number of possible branching paths leading to massive increases of memory usage. As to variant-aware graph model indexing and graphical alignment, a compressed variation graph by merging a genralized Ferrgina-Manzini index (FM-index) [22] encoded subgraph was developed by Siren et al. [23] to generate the novel index GCSA. However, this method is not able to handle a large sequence graph because of the exponentially increase of its index size. To align HTS reads to a collection of genomes, Huang et al.[24] first proposed the Burrows-Wheeler transform-based method BWBBLE. BWBBLE reported aligned reads with a higher confidence than that of the GCSA-based method by compressed representation of multiple genomes and variants from 1090 individuals. It can achieve better efficiency in light of the increasing number of personal sequencing genomes and it shows the potential to improve the subsequent processing pipeline. Eggertsson et.al. [25] proposed a scalable variation-aware graph structure and a novel algorithm Graphtyper to genotype and characterize sequence variation in population genomes. It showed higher accuracy and sensitivity of genotype determination by realigning reads from local genomic regions to the graph. Meanwhile, the variation graph toolkit (vg) developed by Garrison et al. [26] utilized the GCSA2 library [23] to perform read mapping to an arbitrary variation graph and improve accuracy over linear references at the expense of large RAM usage, e.g., the 75 GB RAM theoretical requirement of the GRCh37 linear reference and the variant set produced in the 1000 Genomes Project (1000 GP) phase3 [27]. The development of vg toolkit provides the possibility of a big improvement in post-alignment data analysis algorithms, e.g., realignment, variant calling, haplotype phasing with the gPBWT compression structure [28, 29].

Furthermore, Most of the state-of-the-art generic aligners are implemented in seed-and-extension strategy. The seed extension as a compute-intensive step accommodates the alignment of the read to local sequences surrounding each candidate seed to determine the most likely read position. When determining optimal alignments, approximate string matching in local extension can be crucial, especially for variation graph-based models. The Smith-Waterman (SW) algorithm [30, 31] affected sequence alignment in a siginficant level, and there have been multiple different fast SW applications in various research fields. To be more efficient, vg adopts a graph striped SW algorithm, GSSW [32], to accelerate local alignment via single instruction multiple data (SIMD) implementation. Landau-Vishkin [33] is a banded global SW algorithm with Levenshtein distance penalty scores. Comparing with other global or semiglobal sequence alignment methods, Landau-Vishkin algorithm is an optimization model.

In order to achieve a lower RAM usage during our dynamically construction of pseudo-tree based structure variation tree with different genomic sequence and regional indeded varians in the process of seed extension, we did not load the whole variation set. Also, we developed a read-variation tree alignment algorithm, VARA, utilizing the Landau-Vishkin algorithm and breadth-first traversal on the paths that may consist of the tree nodes and their corresponding variation. Comparing other strategies for tree index-based alignment with reads and a significant number of sequence in the same time, this method can be more efficient and effitive. We intergated VARA into deBGA, a de Bruijn graph-based read aligner, we were able to implement a more comprehensive mapping tool, deBGA-VARA (https://github.com/hitbc/deBGA-VARA).

The benchmark of deBGA-VARA was implement on both one simulation dataset as known variants and the NA12878 sequencing data from 1000 GP. Accroding to the out come, deBGA-VARA was able to reach a higher sensitivity and accuracy than those aligners without considering variation knowledge. For example, deBGA and BWA-MEM [34, 35]. Furthermore, when comparing to other variation-aware aligners such as BWBBLE and vg, our strategy has a faster speed and higher quality with smaller memory footprints, showing that seeding with prior knowledge was not able to obviously improve seeding sensitivity. We hold the belief that such a lightweight global aligner algorithm, VARA, has enormous potential in variant calling and other subsequent biological analyses and could play an important role in prospective genomic studies.

## Methods

### Overview of the deBGA-VARA approach

**Fig. 1:**
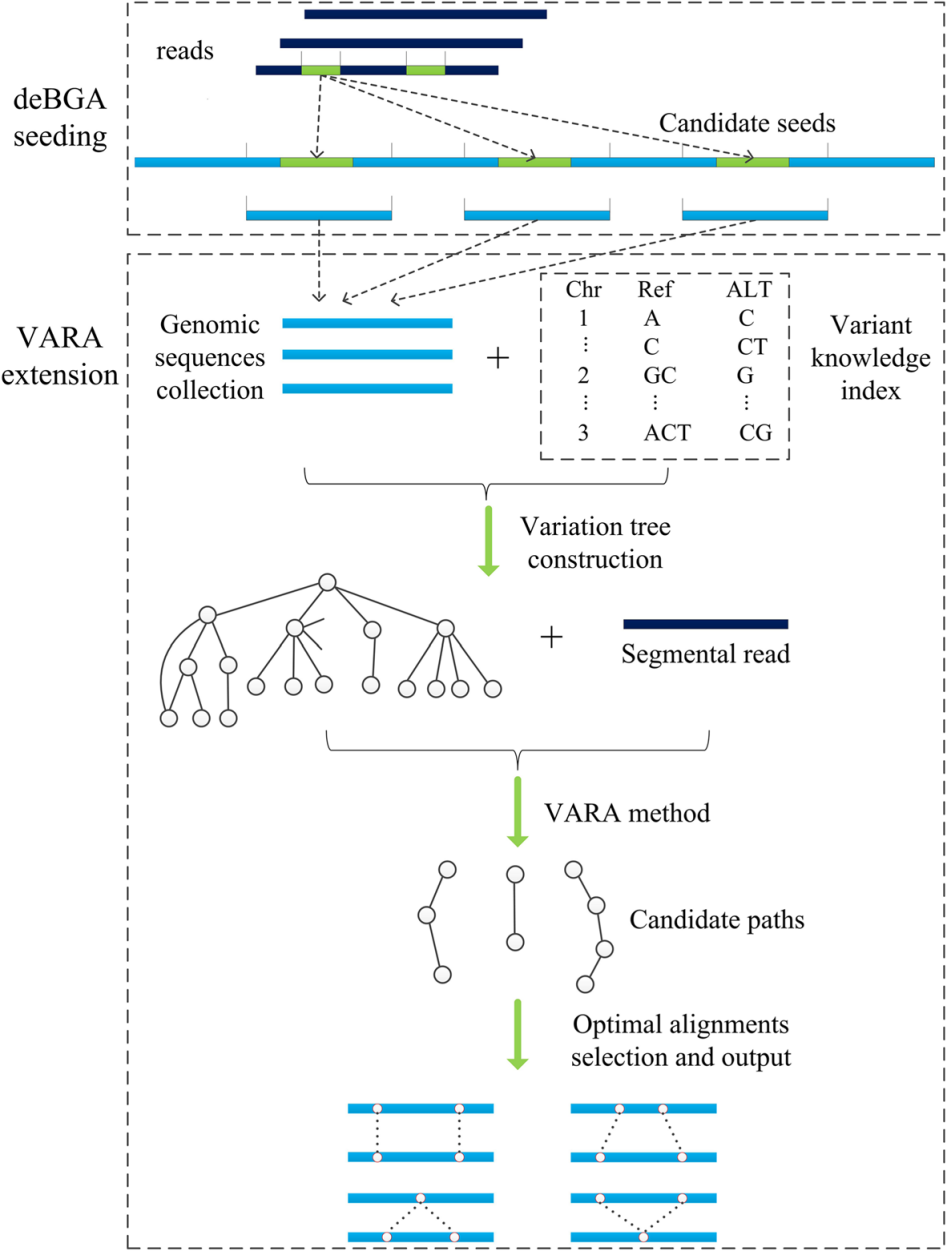
Flowchart of the variation-aware alignment

The deBGA-VARA implements the variation-aware alignment mainly in four steps as follows (a flowchart is in Fig. 1):

1) collect local genomic sequences around the candidate seeds that are generated from the deBGA seeding phase.

2) assemble all the corresponding variant knowledge and construct the variation tree for the novel seed extension.

3) variation-aware alignment between variation tree and the segmental read to generate the candidate paths.

4) optimal alignment selection and output the read mapping result.

### The construction of the variation tree

During genome indexing, the knowledge of INDELs, including variant sequence, variant type, the length and its location, is stored in independent indexing files (ALT-seq) from the original genome index. Also, we intergrated the SNP information into the reference sequence in order to form a variation-aware reference encoded in 4-bit format. In the format mentioned before, both genomic character and its corresponding SNPs can be recorded at one site. During seed extension, to perform alignment with a combination of regional variants, we constructed a pseudo tree-based structure variation tree on-the-fly for indexing genomic sequences and known variation.

**Fig. 2:**
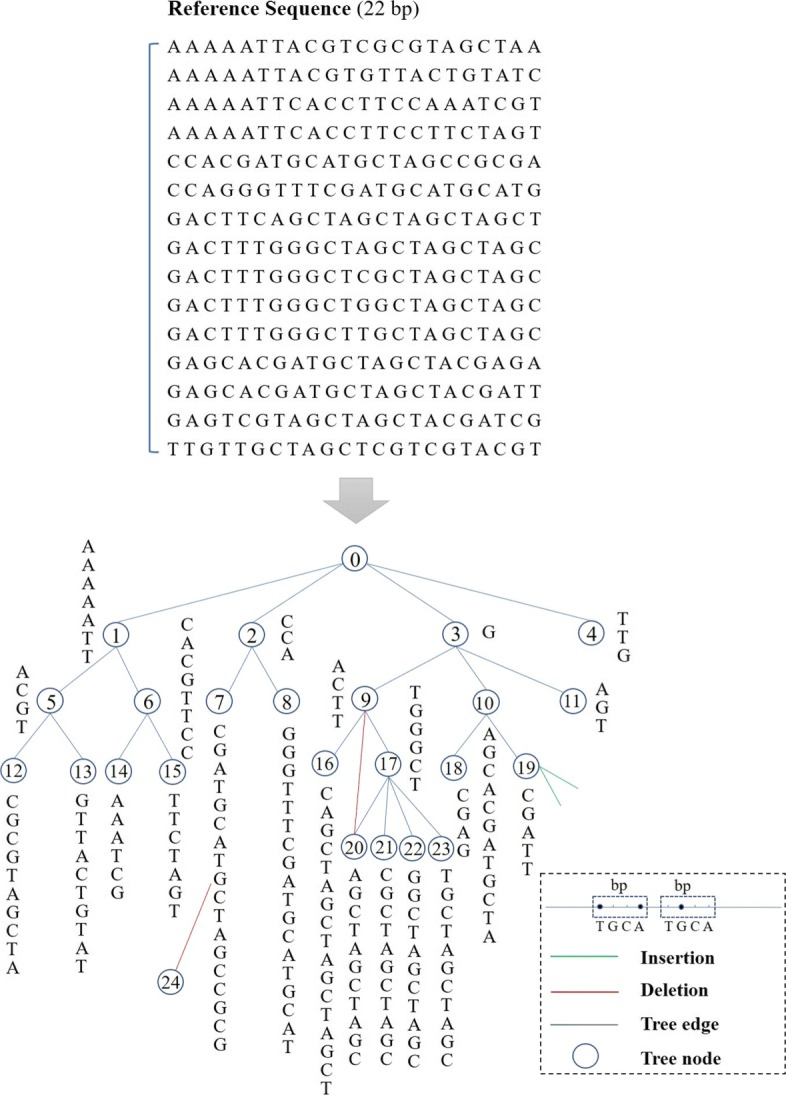
A schematic illustration of the variation tree construction

The sequences for extension are likely to have identical fragments due to so many repetitive regions in human reference. The variation tree collects all local sequences used to construct the tree structure for simultaneous alignment. The nodes in the tree are created alphabetically using the identical part of two or more sequences. One specific node is able to have multiple successor nodes that are located at the subsequent branches, and the node at its next layer is the adjacent successor node. Also, the variants in ALT-seq can connect to the tree node according to its genomic positional relationship to each sequence. Thus, there can be multiple insertions with connections to the same site of an identical node. The long deletion variant can stretch over several nodes, and it is possible to finish at a location beyond the sequence length. Under such circumstance, a new pseudo tree node needs to be created. As shown in Fig. 2, the tree nodes and edges are created based on the identical substrings of 15 ordered reference sequences with length of 22bp. Herein, various variants including SNP, insertion and deletion are connected to the variation tree. Without any variants, the variation tree can become a linear sequence for only one extension sequence.

After building the tree, a path can be generated from a traversal from one node to its successor node or the variant on the way. All possible paths are enumerated in advance according to sequences and correlations between variations and tree nodes. Notably, all paths are independent from each other with a unique ID. There cannot be more than one traversal of identical sequences during subsequent alignment, and some of the nodes can never be reached because of early termination of path traversal due to mismatch occurrence.

### Breath-first traversal on the variation tree

We designed the variation tree-based traversal algorithm VATR for breadth-first traversal of all paths, as shown in Algorithm 1. This method adopts a queue with its basic operations to align a read to a path starting with an arbitrary node. The whole procedure is majorly classified into three categories: i) straightforward node sequence alignment without any variation; ii) traversing nodes including variants and path ID computation; and iii) read exact alignment with a variant sequence (insertion). More specifically, there are also three types of data about to enter the queue: i) the adjacent successors of the current node; ii) newly reached variants; and iii) the ending node of the current variant. Herein, i) *P*_*a*_ is a array that records the path ID; ii) *N*_*a*_ is a array that records the corresponding nodes on the current path; and iii) *Pk*_*a*_ is an array that records cadidate paths. A mismatch-tolerant string comparison strategy was also performed by this method, i.e., it defines an exact match if one character of a read can match a genomic base or SNP at the same locus of reference. Hence, with various types of variation, VATR can accommodate read alignment to variation trees.


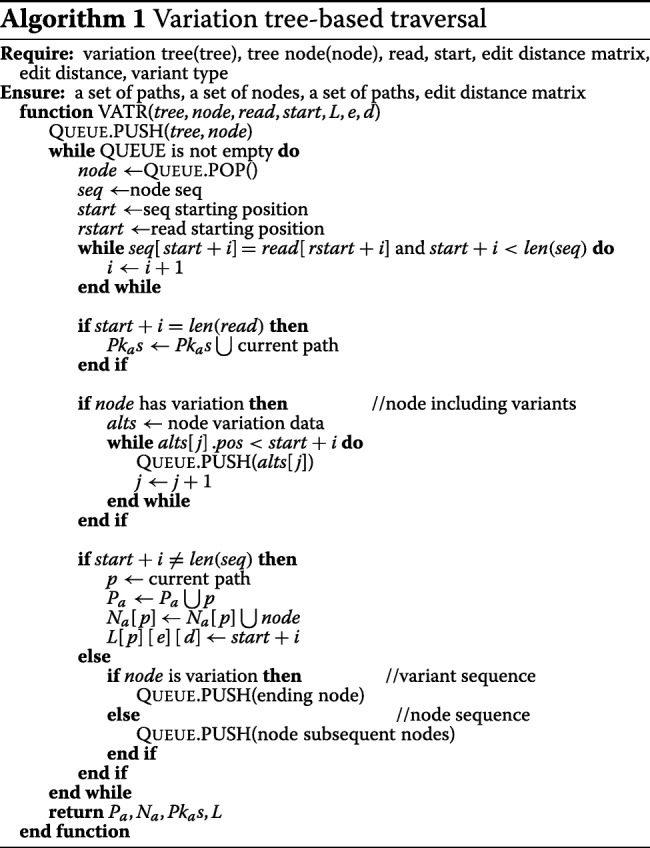


### Variation-aware alignment with generalization of the Landau-Vishkin algorithm

**Fig. 3:**
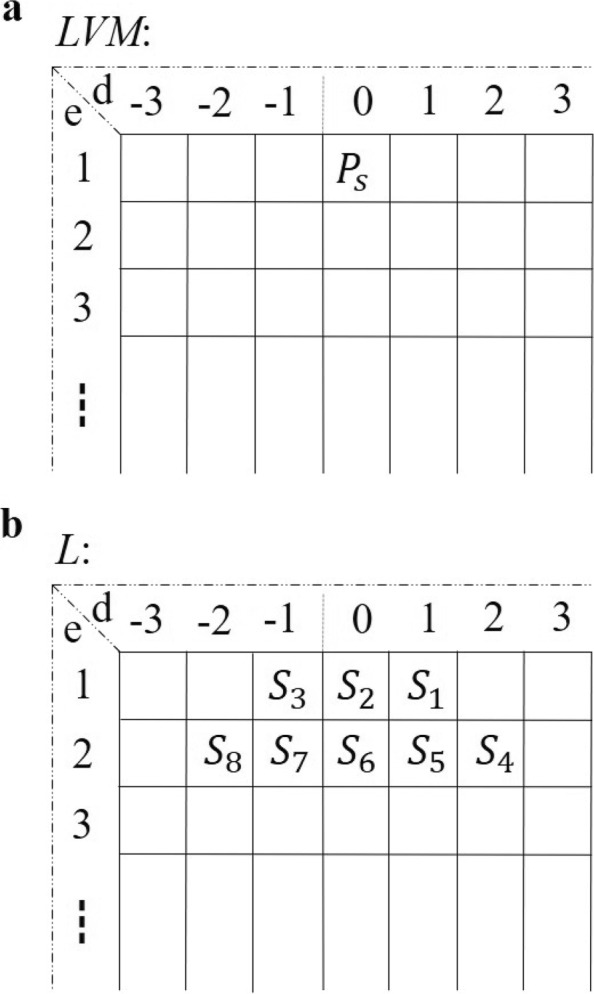
A schematic illustration of the edit distance matrix. **a** The editing distance matrix Landau-Vishkin algorithm. **b** The three-dimensional editing distance matrix

The Landau-Vishkin algorithm improved the dynamic programming strategy over conventional banded global alignment methods and achieved string matching in *O*(*en*) time complexity, where *e* is the maximum edit distance and *n* is the length of the sequence. This method considered the fact that it is not necessary that the algorithm tries to expand the computational cells in a typical dynamic programming matrix (*DPM*) with an editing distance greater than the threshold. Herein, *DPM*[*i*,*j*] is the cell on diagonal *d* of *DPM*, such that *j*−*i*=*d*, where *i* is the row and *j* is column of the matrix. Meanwhile, it maintains an editing distance matrix *LVM*[*e*,*d*], which stores the longest matching distance along current diagonal *d* with edit distance *e* (Fig. 3a). Herein, the *d* in *LVM* also denotes various types of variation including mismatch(zero), insertion(positive value) and deletion(negative value). Landau-Vishkin iteratively calculates cells of *LVM* to exploit optimal alignment with the longest matches, and its recurrence relations are as follows:

1 $$ P_{s} = \max\left\{ \begin{array}{ll} LVM[e-1][d]+1 \\ LVM[e-1][d-1] \\ LVM[e-1][d+1]+1 \end{array} \right.  $$

where *P*_*s*_ is the position query read starts. Based on this principle, we developed a novel global alignment algorithm, VARA, by embedding the traversal alignment process for each path on the variation tree in the Landau-Vishkin framework, as shown in Algorithm 2. The following vital structures store temporary results and contribute to the connectivity of each iteration of traversal alignment.

i) *P*_*a*_*s* denotes two-dimensional arrays that record the ID set of paths. More specifically, *P*_*a*_*s*[*d*] represents the set of paths corresponding to current variation type *d*.

ii) *N*_*a*_*s* is a three-dimensional array that records the ID set of nodes, and *N*_*a*_*s*[*e*][*d*] represents the set of nodes corresponding to different variant types *d* under different edit distances *e*.

iii) *Pk*_*a*_*s* is a two-dimensional array that keeps a record of the ID set of the paths, and *Pk*_*a*_*s*[*e*] represents the set of paths under current edit distances *e*.

**Fig. 4:**
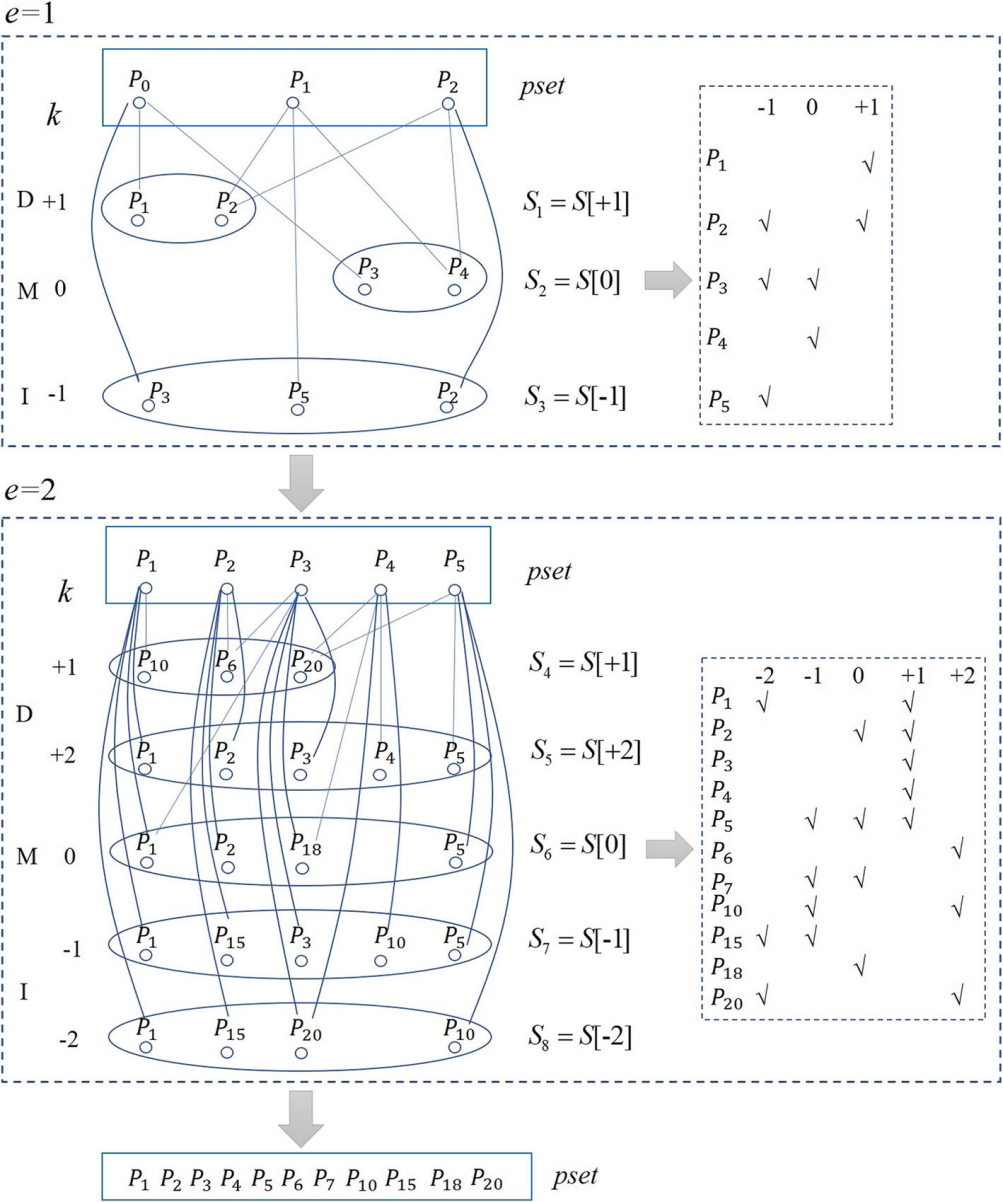
A schematic illustration of the algorithm VARA processing


In VARA, we adopted the VATR algorithm to traverse all paths in the tree index and locate it at the innermost loop in Landau-Vishkin as a substitution for a straightforward string exact match. For a certain path in the current cycle, this method will generate a new path set and its corresponding alignment knowledge. Also, those extension distances of each path with multiple variant types that are matched were collected by us.identical path IDs (Fig. 1) repersents multiple paths. For one path ID, one of its corresponding paths with the longest extension distance will be selected for the next round of traversal alignment. This method serves as the optimal selection strategy in a typical Landau-Vishkin framework. Herein, we changed *LVM* to a three-dimensional matrix (*L*) for storage of variation types and editing distances for multiple paths and continuously updated *L* in VATR for newly generated paths. As shown in Fig. 3b, instead of the conventional matching distance in the *LVM*, each cell records the paths set for current edit distance *e* with the variation *d* (Fig. 4). Moreover, when the entire read is matched under a certain editing distance, VARA will output alignment.

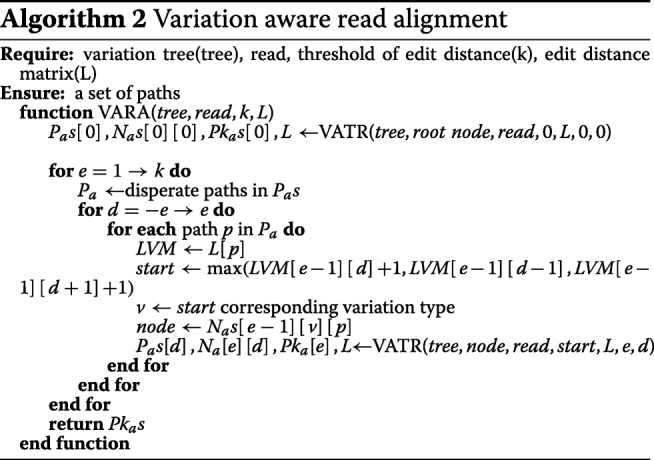


We provide a detailed description of the flowchart of the VARA algorithm (as shown in Fig. 1). Herein, each node represents a path, and the rectangle denotes the currently generated path called *pset*. The ellipse represents the newly generated paths set (*SET*) for different variant types during the current traversal process (each *SET* is given a unique ID, e.g., *S*_1_, *S*_2_, *S*_3_..). For a specific upper limit of edit distance *e*, *d* denotes edit distance within the range from −*e* to *e*, and the positive value, negative value and zero represent deletion(D), insertion(I) and mismatch(M), respectively. This example shows the traversing procedure from the alignment with distance *e* =1 to *e* =2, and the edges in the figure represent the traversal relationship between paths. For instance, *path*2 in the previous period continues to traverse to *path*2 and *path*4 for three types of variations. We can see that traversals from different ancient paths can reach the path with the same ID, and the matrix on the right side is given as a summary of involved paths for different variation types in the current period, e.g., *path*1 is only reached with the deletion type, and *path*3 can be reached with both mismatch and insertion types. After the first round of iteration, the new pset had *path*1, *path*2, *path*3, *path*4 and *path*5. After the second round for distance *e* =2, the new *SET* and *pset* were generated in the same way. This *pset* continued to update as the edit distance increased via rounds of iterations. By this means, we collected all candidate alignments of traversed paths before the end of the entire process.

In order to further increase the extension speed, an optional heuristic strategy to lower the number of traversed paths was also developed by us. More specifically, we terminated the current path traversal before the alignment score exceeded a threshold or there were too many large variants (3 per 100 bp) on the same path. The threshold was based on the fact that it is less likely for a read with a length of 100-300 bps to contain several structural variations simultaneously.

### HTS read mapping with variation

We developed the novel mapping tool deBGA-VARA through integrating VARA into the de Bruijn graph-based aligner deBGA in the phase of extension to accomplish alignment with variation knowledge, only if there are no valid matched seeds for paired-end reads, mainly due to variant occurrence. To be specific, two sides of each candidate seed are semiglobal aligned. For example, we collected all sequences of different regions for the left and right sides of the current seed. After the collection procedure, we used regional variants to built its corresponding variation tree. For seeds only found in a single end, we anchored them to the other end by insertion distance utilizing the strategy in primary deBGA extension. Herein, all seeds from the anchored side underwent VARA analysis except for the seed with the maximum matching length. After such implementations of each end, we merged the outcomes according to their positions and selected the optimal alignment.

**Fig. 5:**
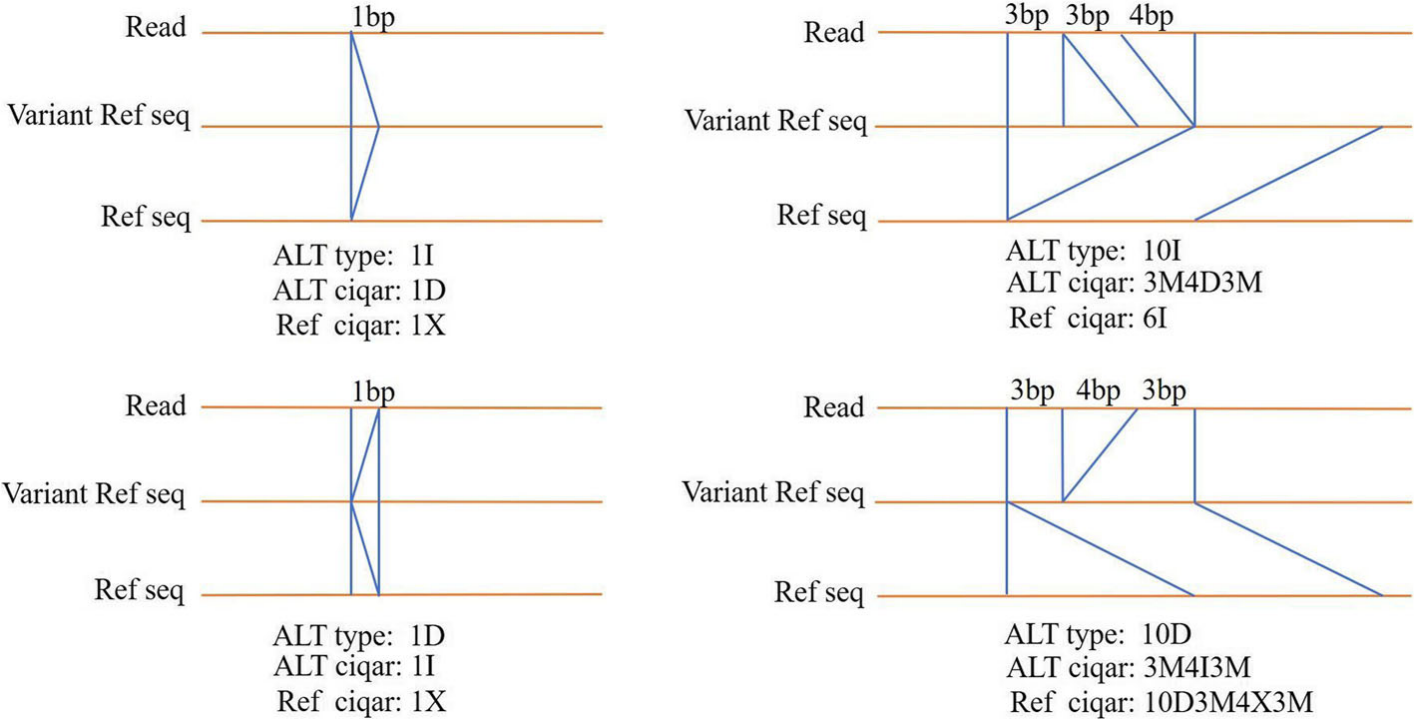
A schematic illustration of the type analysis of cigar restoration

Because of the alignment containing variation, restoring CIGAR to repersent the matching operations in both the read and the original reference sequence was considered to be necessary. We combined the path sequence including the reference and variant knowledge to generate the primary reference-based cigar result. As shown in Fig. 5, it shows the read, the reference sequence with combination of known variation(Variant Ref seq) that is used in VARA approach and the standard reference genome(Ref seq). Herein, the "ALT type, ALT cigar and Ref cigar" indicate variant type, the cigar in alignment that comes out of VARA seed extension and the cigar result based on the original genome respectively. There are four situations in cigar restoration as following.

1) The reference cigar is a mismatch or match if the ALT cigar is 1bp deletion in the context of the 1bp insertion variation.

2) The reference cigar is a mismatch or match if the ALT cigar is 1bp insertion in the context of the 1bp deletion variation.

3) The reference cigar is an insertion if there exists a deletion in the ALT cigar in the context of the insertion variation with the length of more than 1bp.

4) There exists deletion and mismatch in the reference cigar if there exists a insertion in the ALT cigar in the context of the deletion variation with the length of more than 1bp.

Moreover, for each match in the ALT cigar, it needs to restore the cigar according to its corresponding SNP and genomic base.

## Results

The deBGA-VARA was evaluated on two simulation datasets and an HTS dataset to assess its accuracy, sensitivity and speed. Four state-of-the-art aligners, deBGA, BWA-MEM, BWBBLE and vg, were employed for comparison. Herein, BWBBLE was accessed with multiple maximum numbers of mismatches and gaps in the alignment (-n option in the software). All the benchmarks were conducted on a server with 2 Intel E5-2630v3 CPUs at 2.4 GHz (12 cores in total), 512 GB RAM and 48 TB hard disk space (7200 rpm RAID SAS hard disk drive with XFS File System). A single CPU thread (Time-t1) and 8 CPU threads (Time-t8) were used In the benchmarking. The runtime of index loading was excluded for all benchmarked methods.

In order to figure out the performance of the startegy to align reads to regions with different variations, we benchmarked deBGA-VARA with soft clipping statistics. Soft clipping that is represented by character S in the CIGAR specification often appears in the alignment results, indicating the read is not properly aligned to the reference; this is often caused by sequencing errors and various types of variation.

### Benchmarking on simulation datasets

We collected all the variation knowledge of the individual sample NA12878 in Variant Call format (VCF) [36] files that are released from 1000 GP phase3. All these variation records of multiple chromosomes were combined into a single VCF file. Then we merged variants of NA12878 into the hg19 human reference to generate a novel genome dataset named hg19-var. A Mason simulator [37] was used to simulate two datasets of one million Illumina-like pair-end reads (insert size: 500 ± 25 bp) based on hg19-var with different read lengths of 100 bp and 250 bp (Sim-i100 and Sim-i250, respectively). In addition, in order to implement the variation-aware read alignment, we constructed a novel index of the hg19 genome with a combination of variants of this sample.

The results of the simulation dataset are shown in Table 1. Herein, all methods were assessed by several criteria on these two datasets. The runtimes are in seconds (s). Three conclusions can be drawn as follows.

**Table 1:** Statistics on simulated human datasets

Dataset	Aligner	Accuracy % ^a^	Unmapped # ^b^	Soft # ^c^	Time-t1(s)	Time-t8(s)
Sim-i100	deBGA-VARA	99.9	517	1221	114	41
	deBGA	99.9	526	4418	84	39
	BWA-MEM	99.9	0	40368	435	114
	BWBBLE n =2	86.6	972083	0	958	295
	BWBBLE n =6	95.2	92406	0	7378	1074
	vg	99.9	276	23878	10737	1986
Sim-i250	deBGA-VARA	99.9	32	291	212	70
	deBGA	99.9	38	1421	184	61
	BWA-MEM	99.9	0	37830	924	182
	BWBBLE n =6	80.0	398745	0	16483	2387
	BWBBLE n =10	84.4	310725	0	53126	8897
	vg	99.9	12	25451	19164	3761

^a^The mapping accuracy rate.

^b^Number of unmapped reads.

^c^Number of soft clipping reads

i) The deBGA-VARA is several times faster than BWA-MEM, BWBBLE and vg. For example, it is on average fourfold as fast as BWA-MEM. The BWBBLE is almost 250 times slower than deBGA-VARA, even when it is configured to achieve a comparable higher accuracy (e.g., n =10 for 84.4%). Meanwhile, deBGA-VARA and vg achieved almost 100% accuracy for Sim-i100 and Sim-i250; while the accuracy of BWBBLE dropped from 95.2% to 84.4%, even with a large allowable maximum difference (n =10) with the growth of read length. The deBGA-VARA has fewer unmapped reads than deBGA, BWA-MEM and BWBBLE but more than vg, mainly due to that some large known variants are integrated in the seeding phase for vg alignment. Future work should integrate large and complex variants into the seed collection to further increase accuracy and sensitivity. The deBGA-VARA has almost as many unmapped reads as deBGA, mainly because both have identical seeding strategies.

ii) The deBGA-VARA has fewer soft clipping reads than all other methods except BWBBLE (there is no clipping read output of BWBBLE), i.e., the number of soft clipping reads in BWA-MEM is several orders of magnitude larger than that of deBGA-VARA. This finding indicates that reads mapping with known genetic variation can effectively improve the alignment quality. Moreover, there are more clipping reads in vg as the read length increases; however, the quantity of clipping reads in deBGA-VARA decreases greatly.

iii) Vg often requires large amounts of space and time to construct the index, i.e., it requires approximately 320 GB RAM and 20 TB disk space (-X =3 option in the software) to build the xg and gcsa2 index of the human reference genome (GRCh37/hg19) and 200 MB VCF dataset in approximately 40 hours (16 CPU threads). Meanwhile, the deBGA-VARA and BWBBLE require fewer resources to index this identical reference and variation dataset, e.g., 40 GB RAM and 27 mins for BWBBLE and 30 GB RAM and 4 hours for deBGA-VARA. Herein, the index used in the VARA algorithm only requires 2.5 GB memory footprints in alignment.

**Fig. 6:**
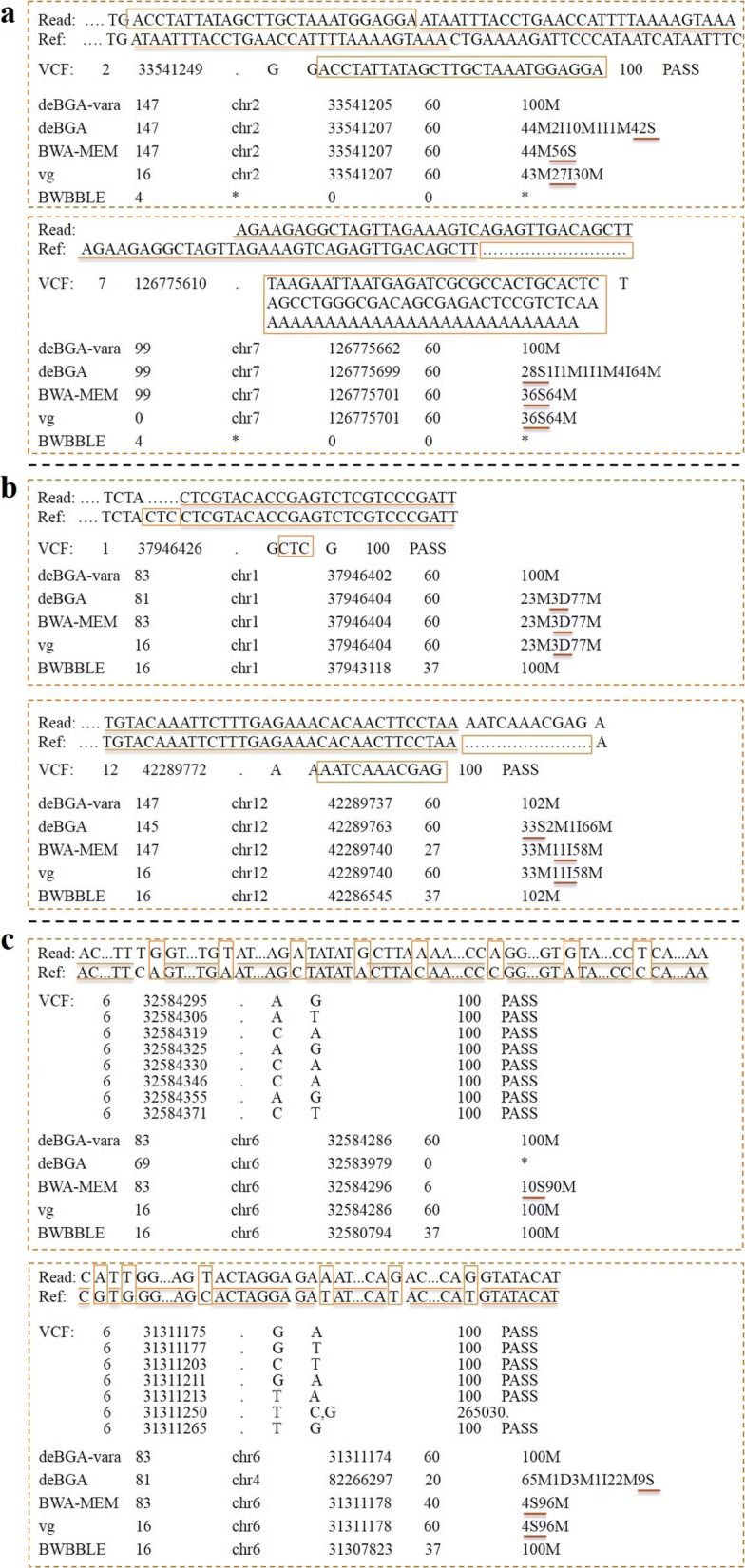
A schematic illustration of read alignments on the HTS dataset and Sim-i100 dataset. **a** Results on simulation dataset. **b** Results on HTS dataset. **c** Results on MHC region

Compared to conventional methods, global alignment with known variation can improve the mapping quality and accuracy. To prove the scalability of deBGA-VARA, we showed six specific examples of read alignments for all aligners with various types of variation in the simulation and HTS datasets (Fig. 6). In each example, the read, reference local sequence, variation in VCF and all alignments are displayed. For each method, there are SAM flags, reference sequence names, positions, mapping quality, and CIGAR strings. It successfully shows two examples of alignments on the Sim-i100 dataset, two examples of alignments on the HTS dataset and the alignments in the MHC region. We can see that the deBGA, BWA-MEM and vg generated poor alignments in the Sim-i100 dataset, e.g., 56 bp and 36 bp soft clipping segments for BWA-MEM and vg, respectively, mainly due to the variation of long insertions or long deletions (Fig. 6a). These two reads in the simulation dataset are unmapped by BWBBLE, which demonstrates that BWBBLE can only integrate small variants (such as SNPs and small indels) but cannot handle relatively larger variations. In this situation, only deBGA-VARA can achieve an accurate alignment because prior knowledge can help to locate the correct genomic sequence on the original reference.

### Benchmarking on the HTS dataset

We also benchmarked deBGA-VARA on the sequencing dataset of the sample NA12878 that was sequenced by Illumina HiSeq 2000 (the read length is 100 bp × 102 bp, and the SRA number is ERR174324). As shown in Table 2, the deBGA-VARA consumes less time than BWA-MEM, BWBBLE and vg on this dataset. However, it is a bit slower than deBGA, due to the time elapsed in variation tree construction and traversal of many paths. Herein, the deBGA-VARA has better sensitivity than BWBBLE with two option settings and has nearly identical sensitivity to deBGA, BWA-MEM and vg. There are more unmapped reads for all methods than those of the simulation dataset.

**Table 2:** Statistics on HTS human datasets

Dataset	Aligner	Mapped % ^a^	Unmapped # ^b^	Soft # ^c^	Time-t1(s)	Time-t8(s)
ERR174324	deBGA-VARA	98.1	37354	72301	565	79
	deBGA	98.1	37479	87653	230	70
	BWA-MEM	99.1	13725	116316	590	78
	BWBBLE n =2	84.5	309005	0	892	219
	BWBBLE n =6	88.8	223736	0	6192	1125
	vg	98.1	28286	91585	13347	2092

^a^The mapping sensitivity rate.

^b^Number of unmapped reads.

^c^Number of soft clipping reads

There are more clipping reads than those in the simulated data set, mainly due to the many sequencing errors and various unknown variation information. A priori variation can contribute to reducing soft clipping reads, i.e., deBGA-VARA has the minimum number of soft clipping reads in the whole genome. Hence, deBGA-VARA maintains high accuracy and sensitivity while reducing soft clipping reads, both on a simulated and sequencing dataset. This method also shows more advantages over both typical HTS read aligners and current variation-aware alignment methods.

Known variation can contribute to determining the correct result but not arbitrary selection of one of the optimal alignments. As a result, this factor can effectively reduce false positives for novel variant discovery. For the known small deletions (3 bp) in the HTS dataset (Fig. 6b), deBGA-VARA and BWBBLE can identify these existing variants but do not consider them to be novel variations and achieve proper alignment. By contrast, deBGA, BWA-MEM and vg offer variants between the read and reference, mainly due to tandem repeats in the region around the known variation, e.g., "CTCCTC". The relatively longer insertion (11 bp) leads to soft clipping alignment in deBGA and known insertion output in BWA-MEM and vg. The deBGA-VARA can provide high-quality alignment results without any clipping segments, while BWA-MEM and BWBBLE have lower mapping quality.

### Benchmarking on the MHC region

We further benchmarked the read alignments produced by deBGA-VARA as well as other state-of-the-art aligners in MHC region. The 4.6-MB extended MHC region (chr6: 28477797-33448354) in human genome is highly reputed by its dense and complex variations, i.e., genomic variations more frequently occur in this region and the combinations of the variations are divergent for various samples. Previous studies [17] have demonstrated that it is still hard to accurately align the reads from MHC region for state-of-the-art aligners with a single reference genome, and the integration of known variations could provide the opportunity to enhance read alignments.

We evaluated the alignment results in MHC region by two aspects. Firstly, we assessed the number of seed hits of the reads to this region. This is critical that, for a given read, the alignment may fail if the aligner cannot find the seed(s) successfully hit the correct locus. We found that the number of correct hits in the reads from MHC region is to some extent lower than that of the reads from the regions having less variations. However, there are still enough hits for them to recognize the correct candidate regions to implement extension alignment, although there are dense variations. Two examples are shown in Fig. 6c, that two reads falling into MHC region respectively have 8 and 6 SNPs, however, deBGA-VARA can still find hits to their grand truth positions. This is mainly due to that the reads are fairly long to span the local region with very dense variations, and its sequencing quality is very high, so that the aligner can still find seed hits in the read parts from the flanking genomic regions with less variations. From this point of view, the introduction of known small variations (e.g., SNPs and indels) in seeding phase could be not as helpful as that of structure variations, since the seeding phase is not seriously affected.

Furthermore, we assessed results of base-level local alignment during the extension phase, which are shown in Table 3 (the statistics on the Sim-100, Sim-i250 and HTS datasets are respectively shown from left to right columns). The deBGA-VARA has the largest number of reads successuly aligned to MHC region, and this number is close to that of grand truth for the two simulated datasets (3862 and 3720 for Sim-i100 and Simi250, respectively). Meanwhile, the deBGA-VARA and BWBBLE align no clipping reads to this area for both of simulation and HTS datasets. There are 3114 alignments on the HTS dataset, which is consistent with the numbers (3855 and 3716) in the simulation data, indicating the effectiveness of read alignment to a region with various complex variants. The numbers of alignments from deBGA, BWA-MEM and vg are nearly identical to each other. The BWBBLE offers few alignments in this region, even with a configuration of n =10 on Simi250. We further investigated the detailed alignments, and found that with variant-aware alignment of deBGA-VARA could better handle the bases spanning genomic variations. For example, for the two reads shown in Fig. 6c, deBGA and BWA-MEM show low mapping quality and soft clipping alignments for both reads. There is a 4 bp clipping segment in alignment of vg for the second read. Only deBGA-VARA can output confidential alignment with much higher mapping quality than BWBBLE and BWAMEM. Notably, the 38th base in the second read is the reference base (’G’) but not the alternative allele (’A’) of the SNP in position 31311211.

**Table 3:** Statistics on MHC region of simulation and HTS datasets

Aligner	Sim-i100 # ^a^	Soft Sim-i100 # ^b^	Sim-i250 #	Soft Sim-i250 #	HTS #	Soft HTS #
deBGA-VARA	3855	0	3716	0	3114	0
deBGA	3750	40	3613	26	3025	96
BWA-MEM	3735	113	3618	93	3058	179
BWBBLE n =2	3070	0	2742	0	2785	0
BWBBLE n =6	3616	0	3157	0	3005	0
vg	3765	53	3620	61	3022	119

^a^number of correct alignments in MHC region on Sim-i100 dataset.

^b^number soft clipping alignments in MHC region on Sim-i100

## Discussion

Local and global alignments play a fundamental role in HTS read mapping and downstream sequence analysis, i.e., the CIGAR outcome of the alignment contributes to variant calling and structural variation detection. However, there is still a high demand to decrease the number of false positive novel variants due to the incorrect alignment results from current mapping methods. Sequences alignment with existing variation can provide a novel strategy to further improve alignment accuracy and mapping quality. We found that integrating the whole set of existing variants into the reference results in explosive growth of graph size in a typical variant graph paradigm. It is non-trivial to handle graph construction and traversal with a large quantity of variation because the possible path enumeration can theoretically be an NP-hard problem. Furthermore, this method often has exponential time and space complexity to reconstruct the index for the continuously updating variation.

Herein, we propose a novel global alignment algorithm, VARA, and developed the mapping system deBGA-VARA by integrating it into a deBGA aligner. We regard deBGA-VARA as a lightweight variant graph-based mapping algorithm. This algorithm combines known variation only to global alignment in the extension step and dynamically constructs pseudo tree-based structures to index variants and sequences in a local genomic scope. The method can be memory scalable due to its limitation of possible variants and paths in VARA. This characteristic can be very beneficial to aligning reads to large variation-aware references. Moreover, the benchmarking results on the simulation and sequencing datasets demonstrated that deBGA-VARA runs much faster than state-of-the-art approaches while maintaining higher sensitivity and accuracy. With its scalability, deBGA-VARA can achieve highly confident alignments both in the whole genome and MHC region. Seeding with variation cannot significantly improve the alignment quality but decreases the mapping efficiency. The deBGA-VARA also showed better results with the increase in read length, indicating its potential for forthcoming sequence analysis.

## Conclusion

It is necessary to integrate large structural variations, e.g., long deletions and insertions, into seed exploration and merging to furtherly increase the quantity of true candidate seeds. Other studies should use this approach in a recurrent framework that will utilize current novel variation outcomes as the input prior knowledge of the next loop of alignment in deBGA-VARA. This strategy can keep improving the quality of novel variants in this iterative process until convergence.

Overall, deBGA-VARA is a promising tool for variation-aware read alignment. This method shows enormous potential in variant calling and complex variation detection for a large population of genomes.

## Data Availability

The deBGA-VARA is available at: https://github.com/hitbc/deBGA-VARA.

## Abbreviations

CIGAR: Compact Idiosyncratic Gapped Alignment Report
deBGA: de Bruijn Graph-based read aligner
DPM: Dynamic programming matrix
FM-index: Ferrgina-Manzini index
GATK: Genome Analysis Toolkit
gPBWT: Graph positional Burrows-Wheeler transform
Smith-Waterman (SW); GSSW: Graph SIMD Smith-Waterman
HBB: Haemoglobin beta
HLA: Human leukocyte antigen
HTS: High-throughput sequencing
INDEL: Insertion and Deletion
MHC: Major histocompatibility complex
PRG: Population reference graph
SNP: Single nucleotide polymorphisms
SIMD: Single instruction multiple data
VARA: Variation-aware read alignment algorithm
VATR: variation tree-based traversal algorithm
Vg: Variation graph
